# Primary signet-ring cell carcinoma of the bladder treated with laparoscopic radical cystectomy: a case report

**DOI:** 10.1007/s13691-023-00648-0

**Published:** 2024-02-06

**Authors:** Yuta Mukae, Kojiro Ohba, Kyohei Araki, Yuichiro Nakamura, Hiromi Nakanishi, Takuji Yasuda, Kensuke Mitsunari, Tomohiro Matsuo, Yasushi Mochizuki, Junji Irie, Ryoichi Imamura

**Affiliations:** 1 Department of Urology and Renal Transplantation, Nagasaki University Hospital, Sakamoto 1-7-1, Nagasaki, Japan; 2 Department of Pathology, Nagasaki Harbor Medical Center, Shinchi-Machi 6-39, Nagasaki, Japan

**Keywords:** Laparoscopic cystectomy, Neoadjuvant chemotherapy, Paclitaxel, Signet-ring cell carcinoma, Urinary bladder

## Abstract

Primary bladder adenocarcinomas comprise 0.5–2% of all epithelial bladder neoplasms. Of these, primary signet-ring cell carcinoma of the bladder is particularly rare, accounting for 0.24% of all bladder malignancies. This tumor is frequently diagnosed at an advanced stage and has a poor prognosis. No standard treatment has yet been established. We here report a patient in whom laparoscopic cystectomy following neoadjuvant chemotherapy was effective. Our patient was a 69-year-old man who had had microscopic hematuria, undergone transurethral resection of a mass in the bladder, and been diagnosed pathologically with a primary signet-ring cell carcinoma of the bladder. No metastases were detected on computed tomography. The patient was treated with a combination of paclitaxel, cisplatin, and gemcitabine prior to undergoing laparoscopic cystectomy. The histopathological diagnosis on this operative specimen was dysplasia and no metastases were detected in the dissected lymph nodes. Complete remission has now been maintained for 9 years.

## Introduction

Primary signet-ring cell carcinoma of the urinary bladder is very rare. The prognosis is generally considered to be poor. In patients without metastases, radical cystectomy is often performed as a local treatment. However, to the best of our knowledge, there are no published reports of laparoscopic or robotic surgery for this type of cancer. In addition, there is no consensus on chemotherapy, including preoperative treatment. Here, we report a patient with primary signet-ring cell carcinoma of the bladder who achieved a complete cure after chemotherapy consisting of paclitaxel, cisplatin, and gemcitabine (PCG) followed by laparoscopic cystectomy.

## Case report

**Fig. 1:**
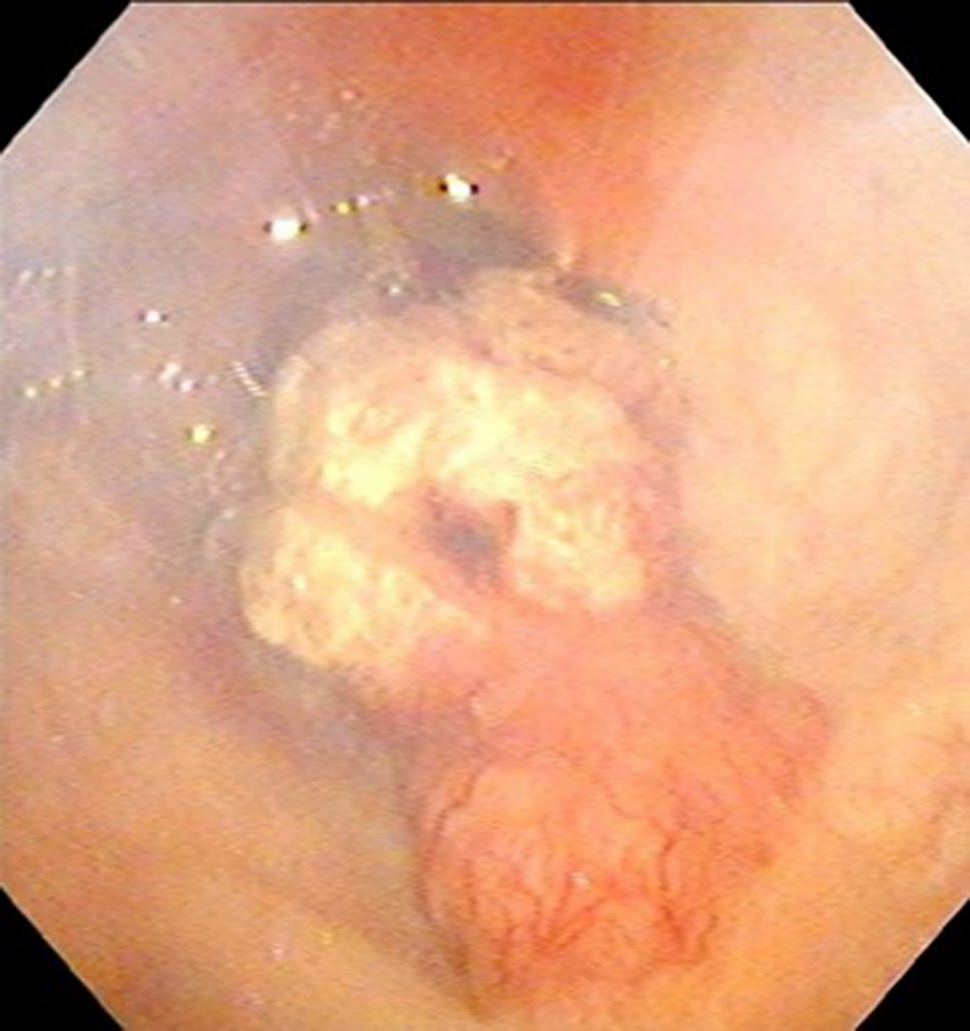
Cystoscopy image. Cystoscopy image showing a non-papillary sessile tumor in the dome of the bladder

**Fig. 2:**
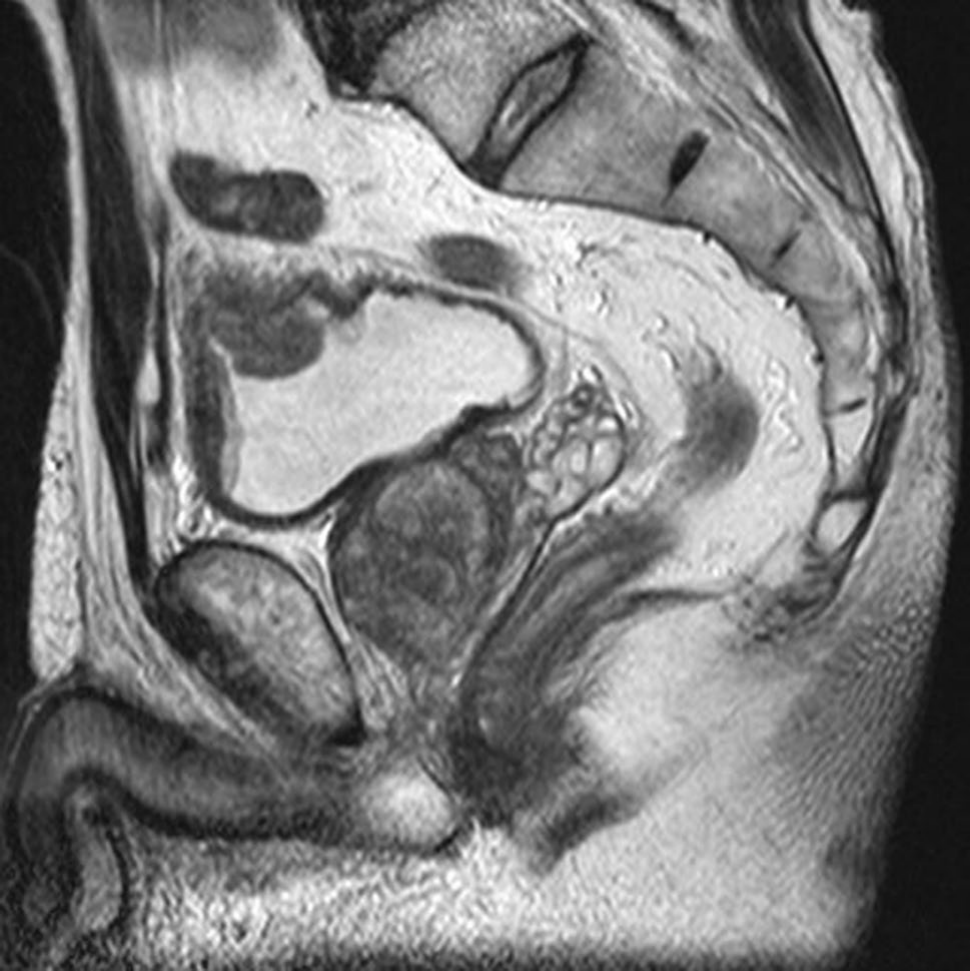
MRI after transurethral resection of bladder tumor. The bladder tumor was suspected to have invaded the muscle layer

**Fig. 3:**
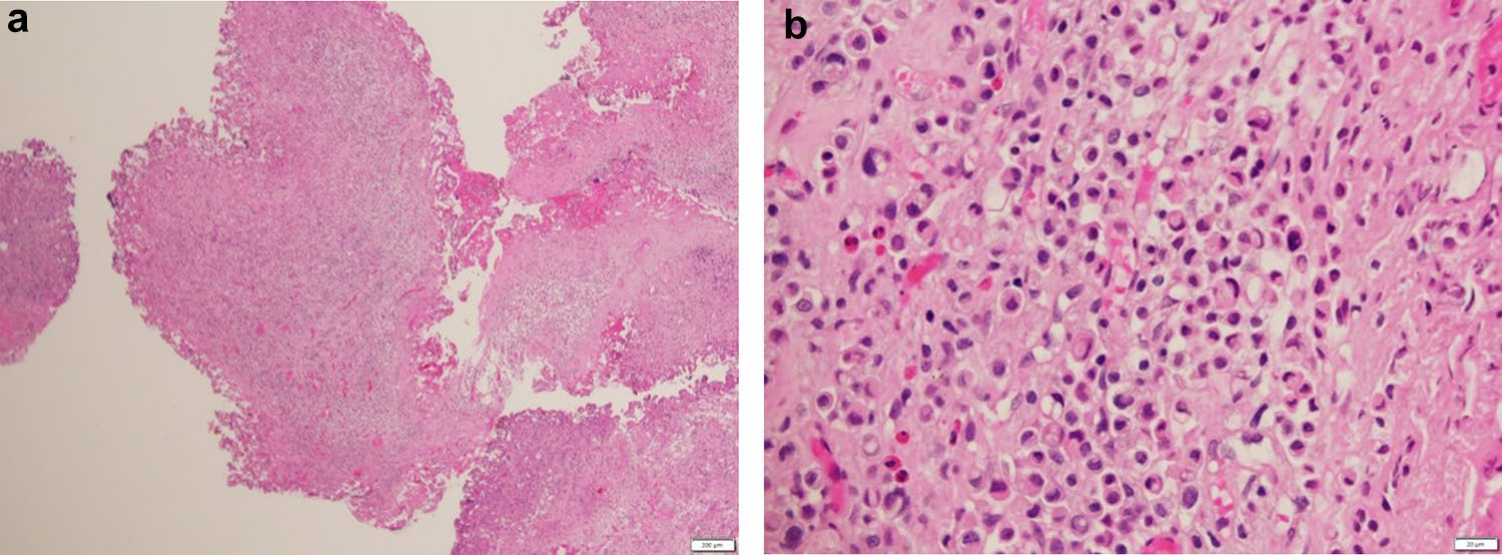
Photomicrographs of the operative specimen. **a** Hematoxylin–eosin staining (40 ×). There is a dense proliferation of tumor cells, which are infiltrating the connective tissue. **b** Hematoxylin–eosin staining (400 ×). There is diffuse proliferation and infiltration of tumor cells containing mucus and unevenly distributed nuclei

A 69-year-old man with no significant medical history was referred to his local hospital because a mass had been detected in his bladder by ultrasonography performed to investigate microscopic hematuria. Cystoscopic evaluation demonstrated a non-papillary sessile tumor in the dome of the bladder (Fig. 1). Urinary cytology was positive and the patient was diagnosed with bladder cancer (cT2aN0M0) on plain magnetic resonance imaging (MRI, Fig. 2). Transurethral resection of the bladder tumor was performed, resulting in a pathological diagnosis of signet-ring cell carcinoma (Fig. 3). Surgery or chemotherapy was offered as treatment options, but the patient requested a second opinion and was referred to our department.

**Fig. 4:**
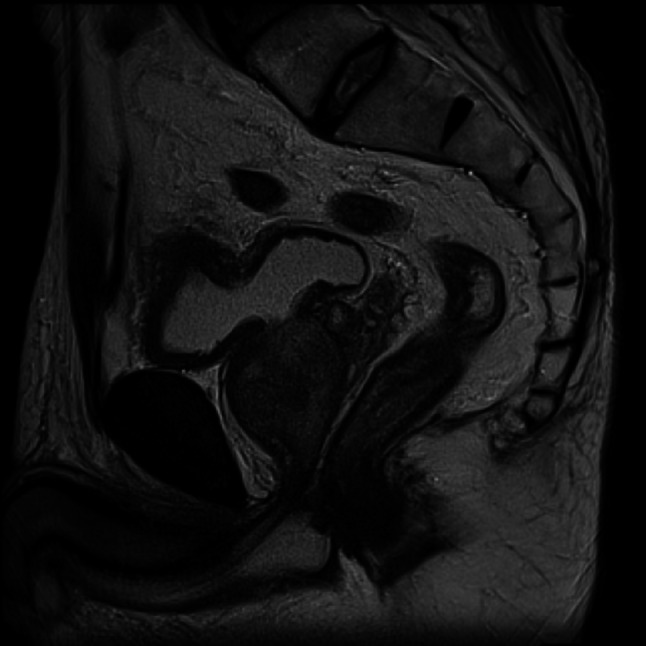
Contrast-enhanced MRI image of the bladder on presentation. Irregular thickening of the wall of the dome of the bladder showing gradual enhancement in a dynamic study. Restricted diffusion was observed

Complete blood count and blood biochemistry were unremarkable, and serum tumor markers carcinoembryonic antigen, carbohydrate antigen 19–9, and squamous cell carcinoma antigen were within their reference ranges. Urine sediment showed microscopic hematuria (> 100 erythrocytes/HPF), and urine cytology was Class I. Plain computed tomography revealed no distant metastases, but lymph nodes of diameter 5 and 8 mm were found in the left and right external iliac artery regions, respectively. Contrast-enhanced MRI showed irregular wall thickening and fat stranding in the bladder dome, suggesting invasion beyond the muscular layer (Fig. 4). No bone metastases were detected on bone scintigraphy. An esophagogastroduodenoscopy found no evidence of malignancy.

**Fig. 5:**
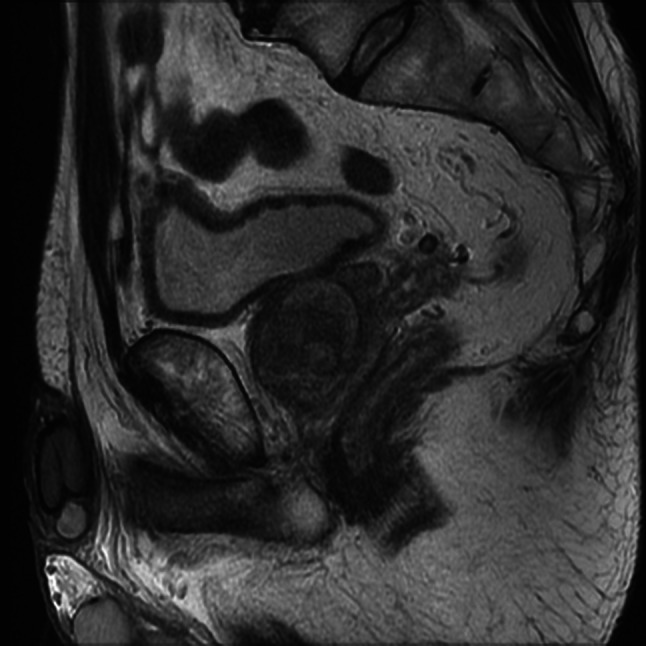
Plain MRI image of the bladder after PCG therapy. The tumor has shrunk and the bladder wall thickening has decreased

Based on the above findings, a diagnosis of primary signet-ring cell carcinoma of the bladder was made and PCG therapy (paclitaxel: days 1, 8 80 mg/m^2^, cisplatin: day 2 70 mg/m^2^, gemcitabine: days 1, 8 1000 mg/m^2^, 28-day cycle) commenced. Grade 4 neutropenia according to the Common Terminology Criteria for Adverse Events (CTCAE; version 5.0) [1] occurred during the first course, necessitating administration of granulocyte colony stimulating factor. Although there were no significant changes in the enlarged external iliac lymph nodes, there was a marked reduction in wall thickening in the dome of the bladder (Fig. 5). Therefore, laparoscopic radical cystectomy (LRC) and ileal conduit construction were performed.

The patient was placed in the Trendelenburg position with open legs. We used a five-port transperitoneal approach. Despite adhesion to the dome of the bladder, it was possible to remove the involved tissue without any major intraoperative complications. Subsequently, pelvic lymph node dissection (common iliac, internal and external iliac, obturator regions, and anterior sacrum) was performed. An ileal conduit was selected for urinary diversion. The operation time was 414 min and the amount of blood loss 1,150 mL. Intraoperative assessment of surgical margins was negative for both ureters and the urethra.

Histopathological examination of the operative specimen revealed anisokaryosis, hyperchromasia, and perturbation of polarity in the epithelium. CK20 and p53 were positive in an atypical part of the urothelium and MIB-1 was unremarkable, leading to a diagnosis of dysplasia. On the serosal side, there was a fibrous area with foamy macrophages and multinucleated giant cells, suggesting that chemotherapy had been effective. Twenty-five lymph nodes were identified, but no metastases were found (ypT0N0).

Nine years after the operation, no recurrences have been identified.

## Discussion

Primary bladder adenocarcinomas comprise 0.5–2% of all bladder neoplasms. Primary signet-ring cell carcinoma of the bladder is particularly rare, accounting for only 0.24% of all bladder malignancies [2, 3]. In many cases, the tumor has already progressed by the time of diagnosis and the prognosis is poor. In a series of 54 cases in Japan, the median age of diagnosis was 61.2 years, with a male dominance of 3:1; additionally, 46% had stage IV disease. The overall survival rate at 2 years was 43%; further, no patients with stage IV disease at diagnosis were alive at 2 years [4].

When diagnosing primary adenocarcinoma of the bladder, including signet-ring cell carcinoma, it is necessary to exclude metastatic tumors, urachal carcinoma, and prostate cancer. In the present case, the tumor was located in the dome of the bladder and urachal carcinoma was initially suspected. However, the tumor was growing into the lumen rather than outside the bladder and there were no urachal remnants or a suprapubic mass. Thus, it did not meet Wheeler and Hill’s criteria for urachal carcinoma [5]. Immunohistochemically, the tumor was positive for AE1/AE3, cytokeratin 7, and cytokeratin 20, indicating that it had originated from the urothelium. Investigations for a primary tumor elsewhere were negative. Therefore, the patient was diagnosed with primary signet-ring cell carcinoma of the bladder.

Some primary signet-ring cell carcinomas of the bladder are associated with high serum carcinoembryonic antigen and carbohydrate antigen 19–9 concentrations. These are reportedly useful biomarkers for assessing prognosis or treatment efficacy [4, 6]. However, in our case, both were within the reference range.

There is no established treatment; however, radical cystectomy is recommended when the tumor is localized [4]. In a report comparing open and laparoscopic radical cystectomy (LRC), although LRC was associated with longer operative time, amount of blood loss, length of hospital stay, need for blood transfusion, amount of narcotic analgesia required, and time to ambulation and regular diet for LRC were superior to open radical cystectomy. There was no significant difference in local recurrence rate or disease-free survival [7]. The range and accuracy of lymphadenectomy associated with radical cystectomy are controversial; however, there is agreement that the number of dissected lymph nodes is an important indicator. It is recommended that 20 or more lymph nodes be dissected [8]. In the present case, 25 lymph nodes were removed, suggesting that the surgical procedure was adequate. Because LRC has few complications, an additional advantage is that chemotherapy can be commenced immediately after surgery if considered necessary. To the best of our knowledge, there are no published reports on the patient performed with LRC for bladder signet-ring cell carcinoma. Our patient was discharged 23 days postoperatively without major complications and has been free from recurrence ever since. Robot-assisted surgery is now available and there is a shift toward minimally invasive surgery. We believe that reporting this case of complete cure after minimally invasive surgery will be helpful.

Various chemotherapy regimens have been reported, including gemcitabine with cisplatin and the combination of methotrexate, vinblastine, doxorubicin, and cisplatin, both of which are prescribed for standard urothelial carcinoma. Use of drugs used for advanced gastric cancer, such as S-1 (tegafur/gimeracil/oteracil) has also been reported. However, there is currently no standard chemotherapy regimen. Furthermore, the usefulness of NAC for UC with variant histology is not clear. In regards of neoadjuvant therapy, its usefulness for UC with variant histology as well as pure UC has been reported [9]. We considered that NAC is also effective for this case with signet ring cell carcinoma, which is one of variant of UC. Table 1 shows the regimens used in 12 cases of bladder signet-ring cell carcinoma treated with chemotherapy [4, 6, 10–18]. However, these reports were not about neoadjuvant therapy, but about adjuvant therapy or systemic therapy for metastatic disease. In addition, these reports were all about regimens that urologists do not normally use or associated with severe adverse events. We selected PCG therapy as neoadjuvant chemotherapy for our patient because of its high grade compared with the usual urothelial carcinoma and a report that PCG therapy prolonged overall survival compared with gemcitabine with cisplatin in patients with advanced urothelial carcinoma [19]. Furthermore, since we are accustomed to usage paclitaxel, we thought that it would be possible to deal with adverse events if paclitaxel was added to gemcitabine and cisplatin. The reported regimen consisted of paclitaxel 80 mg/m^2^ (days 1 and 8), cisplatin 70 mg/m^2^ (day 2), and gemcitabine 1000 mg/m^2^ (days 1 and 8) in 3-week cycles. However, in this case we adopted a 4-week cycle because of adverse events. Although the patient required granulocyte colony stimulating factor for management of neutropenia, two courses were administered. Histopathological examination of the operative specimen confirmed the efficacy of the treatment. The optimal number of courses of preoperative chemotherapy remains controversial. However, we have identified no postoperative recurrences in our patient, suggesting that PCG therapy is an effective treatment for primary signet-ring cell carcinoma of the bladder.

**Table 1:** Characteristics of 12 cases of bladder signet-ring cell carcinoma treated with chemotherapy

Authors	Age	Sex	Operation	Regimen	Follow-up (month)	Outcome
Hirano et al. [9]	65	M	None	Carboplatin (intra-arterial)	44	Alive
Akamatsu et al. [4]	55	F	None	S-1	5	Dead
Akamatsu et al. [4]	76	M	None	S-1 + carboplatin	Not reported	Dead
Michels et al. [6]	81	M	None	Gemcitabine + carboplatin Capecitabine	12	Dead
Shringarpure et al. [10]	48	M	Radical cystectomy	Gemcitabine + cisplatin (A)	12	Alive
Singh et al. [11]	62	M	Partial cystectomy	Capecitabine + oxaliplatin	32	Dead
El Ammari et al. [12]	51	M	Radical cystectomy	Gemcitabine + cisplatin (A)	22	Dead
Hamakawa et al. [13]	53	M	Radical cystectomy	S-1 + cisplatin (A)	90	Alive
Pugashetti et al. [14]	71	M	Radical cystectomy	FOLFOX	12	Alive
Di Maida et al. [15]	57	M	(Radical prostatectomy)	Gemcitabine + paclitaxel	4	Dead
Hinduja et al. [16]	66	M	(Transurethral resection)	FOLFOX	10	Alive
Benerjee et al. [17]	42	M	Partial cystectomy	ddMVAC (A)	6	Alive

*A* adjuvant chemotherapy, *ddMVAC* dose-dense methotrexate, vinblastine, doxorubicin, and cisplatin, *FOLFOX* 5-fluoro-uracil, folinic acid, and oxaliplatin, *S-1* tegafur/gimeracil/oteracil

In conclusion, this patient with primary signet-ring cell carcinoma of the urinary bladder, which generally has a poor prognosis, was administered PCG as neoadjuvant therapy, then underwent LRC and achieved a prolonged complete response.
